# Mental practice with interactive 3D visual aids enhances surgical performance

**DOI:** 10.1007/s00464-017-5459-3

**Published:** 2017-03-10

**Authors:** Marina Yiasemidou, Daniel Glassman, Faisal Mushtaq, Christos Athanasiou, Mark-Mon Williams, David Jayne, Danilo Miskovic

**Affiliations:** 1 Leeds Institute of Biomedical and Clinical Sciences, University of Leeds, St James University Hospital, Clinical Science Building, Beckett street, Leeds, LS9 7TF UK; 2 School of Surgery, Health Education Yorkshire and the Humber, University of Leeds, Willow Terrace Road, Leeds, LS2 9JT UK; 3 School of Psychology, Faculty of Medicine & Health, University of Leeds, Leeds, LS2 9JT UK

**Keywords:** Mental practice, Surgical training, Anatomical models

## Abstract

**Background:**

Evidence suggests that Mental Practice (MP) could be used to finesse surgical skills. However, MP is cognitively demanding and may be dependent on the ability of individuals to produce mental images. In this study, we hypothesised that the provision of interactive 3D visual aids during MP could facilitate surgical skill performance.

**Methods:**

20 surgical trainees were case-matched to one of three different preparation methods prior to performing a simulated Laparoscopic Cholecystectomy (LC). Two intervention groups underwent a 25-minute MP session; one with interactive 3D visual aids depicting the relevant surgical anatomy (3D-MP group, n = 5) and one without (MP-Only, n = 5). A control group (n = 10) watched a didactic video of a real LC. Scores relating to technical performance and safety were recorded by a surgical simulator.

**Results:**

The Control group took longer to complete the procedure relative to the 3D&MP condition (p = .002). The number of movements was also statistically different across groups (p = .001), with the 3D&MP group making fewer movements relative to controls (p = .001). Likewise, the control group moved further in comparison to the 3D&MP condition and the MP-Only condition (*p* = .004). No reliable differences were observed for safety metrics.

**Conclusion:**

These data provide evidence for the potential value of MP in improving performance. Furthermore, they suggest that 3D interactive visual aids during MP could potentially enhance performance, beyond the benefits of MP alone. These findings pave the way for future RCTs on surgical preparation and performance.

Mental Practice (MP) can be defined as the cognitive practice of a task without overt physical movement. The approach is used routinely in sports and performing arts to enhance performance [1–5]. More recently its potential has been explored for explicit learning of surgical skills [6], but there remains a number of questions about MP’s effective implementation in the field of surgery, as studies had mixed results [7–15].

MP is a demanding process involving image generation, maintenance, inspection and transformation [16–18]. In MP, an image can be generated from direct perceptual information [19] or from resurfacing information previously stored in long-term memory [20]. A potential explanation for the lack of agreement amongst studies on the benefits of MP is that the quality of the mental image generated can vary substantially across individuals [21, 22] and is, of course, altered by one’s experiences [23, 24].

The cognitive resources required for image retention are notably high [25–27], requiring increased amounts of sustained attention [19, 28, 29] to prevent the generated image from fading in milliseconds. This is achieved mainly through the stimulation of visual memory through processing the properties of the objects found in the generated image [18]. This involves morphological assessment [20] and image manipulation [19, 29] (such as rotation, transportation [16, 30] or image reconstruction [31]). We speculated that cognitive demands may constrain the quality of the MP process and thus, impact on the benefit that could be accrued from this approach. Thus, we hypothesised that the provision of additional resources, such as interactive 3D models of the task-relevant anatomy, during MP should facilitate the process and subsequently impact on performance in trainee surgeons.

## Materials and methods

### Ethical approval

After consultation with the Research and Development (R&D) department of Leeds Teaching Hospitals, it was advised that approval by an NHS research ethics committee or the R&D department was not required. The study received departmental approval by the research lead of the surgical Clinical Service Unit (CSU).

### Participants

Twenty junior specialty trainees (core trainees and early registrar years; postgraduate years 2–4) were recruited for this study. The surgeons were case-matched 1:1:2 (MPO: 3D&MP: Control) based on the following variables: number of real laparoscopic cholecystectomies conducted as primary surgeon and number of times they had used the same virtual reality simulator in the past (Table 1). All participants had seen and assisted in Laparoscopic Cholecystectomy (LC) operations but had not performed more than 15 as primary surgeons. Twice as many participants were allocated to the control group in order to increase statistical power [32]. This is particularly desirable if the cost for including additional control is minimal [33]. Specifically, asking individuals allocated to the control condition to review a pre-prepared didactic video bears no additional cost to the study and is a method that these surgical trainees were familiar with as it is used during teaching sessions of surgical skills within the area.

**Table 1:** Trainees’ experience at baseline

Group	3D	MPO	CG
No of LC as primary surgeon (median, range)	7 (3–14)	4 (0–15)	5.5 (2–12)
No of times simulation was used (median, range)	1–5 (0–more than 10 cases)	0 (0 to 6–10 cases)	0 (0 to more than 10 cases)

### 3D model

**Fig. 1:**
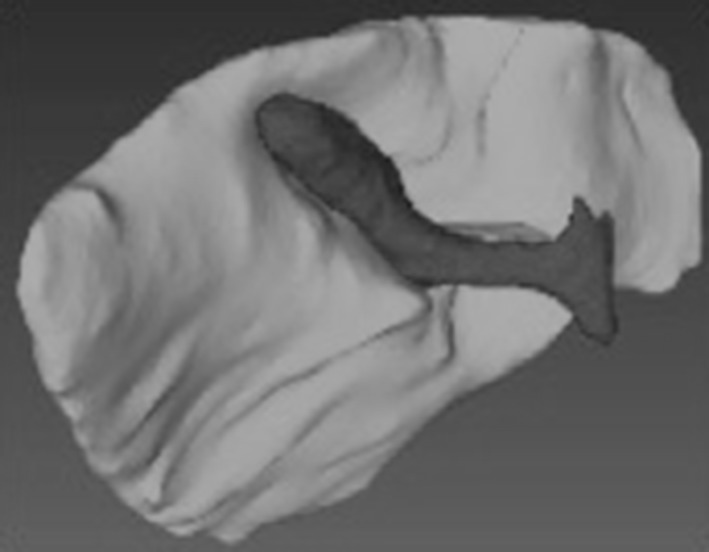
Interactive 3D model

The 3D model was reconstructed from an anonymised Computed Tomography (CT) transferred through a Compact Disc (CD) in Digital Imaging and Communications in Medicine (DICOM) form, to “in-house” 3D reconstruction software (VolumeViewer, University of Leeds). The 3D model was created through manual reconstruction to “match” the VR-simulated images of a normal anatomy gallbladder, biliary tract and vascularity (Fig. 1). The model was then exported onto open source visualisation software (MeshLab) and underwent minor contouring.

### Intervention

**Fig. 2:**
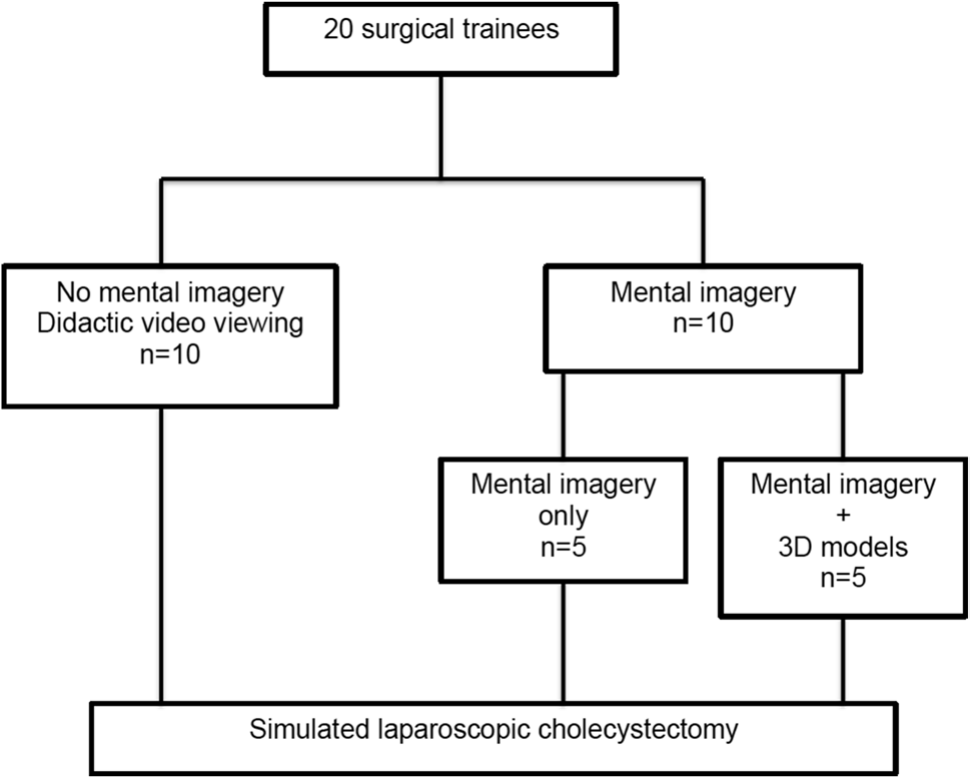
Flowchart of study design

The Control Group (n = 10) was exposed to a didactic real time video of a LC, whilst two intervention groups (3D group; n = 5) and Mental Practice Only (MPO; n = 5) underwent a single 25-min Mental Preparation (MP) session in the presence of a facilitator. For the 3D group, an interactive 3D model of the relevant surgical anatomy (Fig. 1) was incorporated into the MP process (Fig. 2).

Prior to the commencement of MP, the experimenter, acting as a facilitator, provided demonstrations of verbalised mental preparation to the participants. In addition, the opportunity to train on how to use the VR simulator (LAP Mentor, Simbionix, Cleveland, OH, USA) was provided. The participants were taught practical aspects of simulation usage, such as how to select a surgical tool and where the diathermy pedals are located. The trainees did not require additional training on how to perform a laparoscopic cholecystectomy as they had previously assisted or/and performed laparoscopic cholecystectomies.

The intervention groups (MPO and 3D) were given an excerpt from a surgical textbook [34] containing a step-by-step breakdown of a LC and were asked to “visualise” and “feel” the operation. Participants allocated to the 3D group were taught how to use the rotation and zoom-in/-out tools of the visualisation software. In addition to MP, they were instructed to inspect the 3D anatomy on the virtual model for each step of the procedure.

All groups, after undergoing the appropriate preparation process, proceeded to perform a simulated laparoscopic cholecystectomy on a VR simulator. Performance (Instrumental Tip Path Length [PL], Number of Movements [NOM] and Time To Extract Gallbladder [TTGB]) and safety metrics (Number of Non-Cauterised Bleedings [NCB], number of Perforations [Per], number of Damage to Vital Structures [DVS]) automatically provided by the simulator, were recorded. We chose these performance metrics because previous research has demonstrated that they have predictive validity between experts and novices [35]. There was no previous validation of the safety metrics recorded in this study; however, these metrics were selected ahead of other measures on the basis that poor performance on these would have a clear impact on patient wellbeing if these operations were to be performed in a real clinical setting.

### Statistical analysis

The performance and safety metrics were tested for departures from normality using the Shapiro–Wilk test before being subjected to a One-Way ANOVA or a non-parametric Kruskal–Wallis test as appropriate. The Shapiro–Wilk test indicated that the performance metrics were normally distributed (Time p = .87, NOM p = .67, PL p = .93). However, safety measures were demonstrated to have a non-normal distribution (p < .001). As a result of the normality testing, one-way ANOVA was used for the performance metrics and Kruskal–Wallis testing was used for the safety metrics. For the ANOVA, when a significant difference for a main effect (*p* < .05) was found, Bonferroni-corrected post hoc comparisons were performed. For brevity, only statistically significant post hoc comparisons are reported. We report partial eta squared values (*η*_*p*_ *²*) to indicate the effect size. An estimate of the effect size w² is reported (H/N). Analysis was carried out using IBM SPSS® version 22 (IBM, Armonk, NY) and GraphPad Prism 6.0 (GraphPad Software, Inc., California, USA).

In addition to this analysis, we also computed the Bayes factor (BF) for each of our outcome measures [36] (Table 2) using JASP Statistics (v 0.7.5.6) [37]. This approach has several advantages relative to traditional null hypothesis significance testing (p values) [38] and we report it here for two reasons. First, BFs allow us to determine whether the obtained evidence favoured the alternative hypothesis (there is a difference in outcome measures for our three conditions- with a BF > 1), the null hypothesis (BF < 1) or neither (BF equal or close to 1). This is particularly important for understanding the data when p values are greater than our significance threshold of 0.05 [39]. Second, it is particularly advantageous to use the BF when results need to be directly comparable with future work. This is because the obtained value represents a ratio of the probability of the null and alternative hypotheses and thus it is not biased by sample size [40]. To interpret the strength of the BFs, we considered a BF ranging from 1 to 3 as barely consequential evidence and a BF greater than 10 as “strong evidence” [41].

**Table 2:** Relative evidence for H1 compared to H0 H_1_ (BF_10_)

Outcome Measure	BF_10_
Time	17.71
Total number of movements	52.42
Path length	12.35
Number of perforations	0.51
Frequency of non-cauterised bleeding	0.63
Damage to vital structures	0.45

## Results

### Bayes factors

Examining the BF values indicated that there was strong evidence that the data obtained were 17.71, 52.42 and 12.35 (Table 2) more likely under the alternative hypothesis (that there is a difference across conditions) for Time, Total Number of Movements and Path Length, respectively, than the null hypothesis. This analysis also shows that there was more evidence for the null hypothesis than the alternative for all three safety metrics.

### Performance metrics

**Fig. 3:**
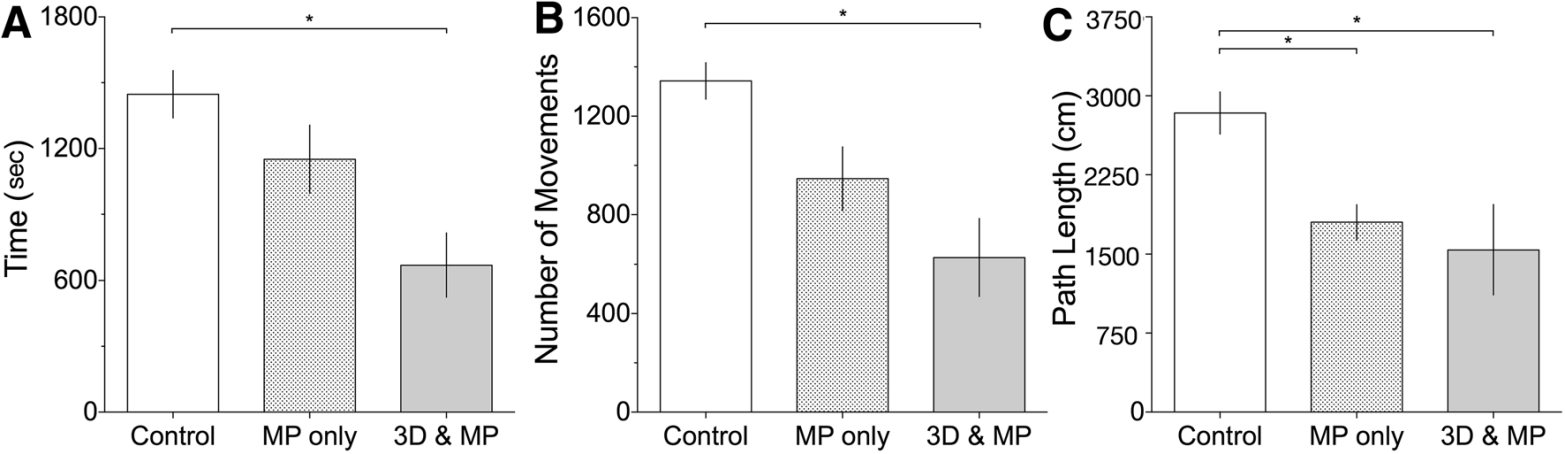
Performance metrics (*error bars* represent ± 1 SEM)

Results from our frequentist analysis were consistent with the BF calculations. We found that performance differences across groups (Fig. 3) showed a main effect in the amount of time taken by participants to complete the simulated LC (F(2,17) = 8.77, p = .002, *η*_*p*_ *²* = 0.51), with the Control group (M = 1447, SD = 341) taking significantly longer (p = .002) relative to the 3D & MP group (M = 670, SD = 326). Similarly, the NOM was also significantly different across groups (F(2,17) = 11.57, p = .001, *η*_*p*_ *²* = 0.58), with the 3D & MP groups (M = 627.2, SD = 352) making fewer movements relative to controls (p = .001). For PL, a significant main effect was found (F(2,17) = 7.57, p = .004 *η*_*p*_ *²* = 0.47) and we observed that the control condition (M = 2837, SD = 633) led to longer distances covered in comparison to the 3D & MP condition (M = 1540, SD = 957, p = .008) and the MP-only condition (M = 1800, SD = 370; *p* = .038).

### Safety metrics

**Fig. 4:**
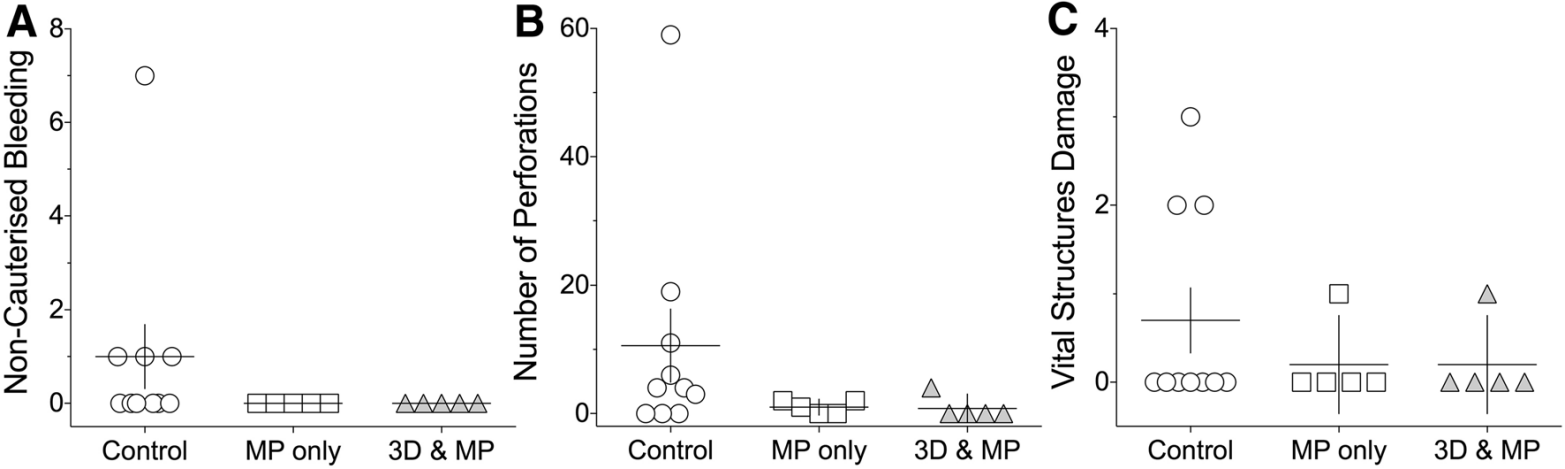
Safety metrics (error *bars* represent ± 1 SEM)

For the safety measures (Fig. 4), no statistically significant difference was found in the frequency of the damage to vital structures (H(2) = 0.63, p = .68, ω^2^ = 0.03). The comparisons for non-cauterised bleeding (H(2) = 4.71, p = .13, ω^2^ = 0.24) and number of perforations (H(2) = 4.8, p = .082, ω^2^ = 0.24) showed marginal trends but did not reach the statistical significance threshold.

## Discussion

There is a long history of using mental practice to improve performance in sports and arts. Whilst recent work has suggested that this approach could be adapted for skill acquisition, evidence remains equivocal [7, 8, 10]. We suggest that, because the ability to produce a mental image varies across individuals [42], this could potentially account for differences across studies. It is also known that MP is a demanding process [17, 18, 43], requiring a number of cognitive processes to work in concert [25–27]. We speculated that providing support for MP might enable trainee surgeons to maximise the benefit of this approach.

The present study therefore applied a novel approach and developed interactive 3D visual models. The hypothesis was that this would alleviate the cognitive load of producing and maintaining a virtual image and standardise the quality of the image produced amongst individuals, which we hypothesised would subsequently lead to better surgical performance. When this approach was compared with pre-procedural preparation using didactic video viewing, there was an indication from our data that this may enhance the assessed surgical performance metrics. Conversely, mental imagery alone appeared to enhance only path length when the same comparison applied.

Safety metrics (damage to vital structures, non-cauterised bleedings, liver perforations) were found to be similar in the three groups. Adverse events, with the exception of two outliers, were rare occurrences (Fig. 3), which may not have been the case if the participants recruited had been novices (i.e. have not performed the operation as a primary surgeon).

The concept of the current study is novel—no trial to date has combined a 3D model with mental practice. However, several trials have assessed the effect of mental rehearsal without the use of additional aids [7, 8, 10, 44, 45] with conflicting results. The MPO group in our study improved in only one metric (PL) when compared to the control group. Other metrics, such as NOM, showed a trend and a greater sample size study may have demonstrated a more conclusive enhancement of performance.

Similarities can also be found in the methodology described in the relevant literature. For instance, the duration of the MP sessions is similar with previous studies assessing the acquisition of surgical skills after MP [7, 8, 10, 13, 45]. Similarly to the current study, Mulla et al. [11] and Eldred-Evans et al. [45] used a step-by-step breakdown of the procedure while Sanders et al. [10] used a textbook to facilitate the consequent MP process. Other authors have applied training sessions on how to perform a laparoscopic cholecystectomy, but this was necessary because they recruited medical students who were not familiar with the performed procedure [10–14, 45]. The current study recruited advanced beginners who were familiar with the technique as surgical assistants or through performing the operation as primary surgeon.

It should be noted that there are limitations to this study. Firstly, this was an exploratory, case controlled (rather than randomised trial) with a small sample size of 20 participants. The limited sample size increases the possibility of making a type II error. Relatedly, the use of eta square calculation may overstate the effect size due to the small number of participants in the intervention groups (n = 5). As such, the generalizability of these results may be limited due to the small sample size of this study. Nevertheless, this exploratory finding does provide an interesting avenue for a future, larger scale, statistically high powered RCT. To inform future work, using an average obtained effect size from our performance metrics (*η*_*p*_ *²* = 0.52), we computed (using G*Power 3.1.9) [46] that a minimal sample size of 42 would need to be adopted to achieve 80% power (1-β error probability). We add the caveat that the eta square may be overstating the effect size in such a small sample so the sample size may need to be considerably larger. We anticipate that future studies will contribute information that will enable more accurate estimates.

It is also noteworthy that, unlike previous studies, in which medical students were recruited [10–14, 45], only surgical trainees participated—a more representative sample of the target population. Recruitment of inappropriate participants in education studies has previously been highlighted by the Association for Surgical Education (ASE) who concluded that recruiting medical students is not an appropriate method for validating educational methods that are targeted at surgical trainees [47].

To address the issue of individual differences in MP, some previous studies tested the baseline ability of the participants to perform MP in order to ensure equality of the comparative groups [12, 48]. In our study, we adopted an alternative approach (as our priority was to facilitate group level performance rather than attenuate individual differences) and instead standardised the presentation of the visual model, the presence of a facilitator and the provision of a textbook excerpt throughout the MP session.

Specialties such as orthopaedic and vascular surgery have been using imaging 3D reconstruction to make treatment decisions [49–51]. However, the anatomical model viewing process has not been employed in a systematic manner, thus providing a different experience for individual surgeons. Furthermore, three-dimensional patient-specific models have not been explored for didactic value in non-experts. Mental imagery provides an ideal platform for both a systematic approach and for boosting the potential didactic effect of anatomically variant models. This combined approach of mental imagery and anatomically variant anatomical models may be applied both in the pre-operative preparation of expert surgeons prior to complex surgical procedures or in didactic sessions for novices. For instance, minimally invasive total mesorectal excision complexity has been shown to be associated with patient (e.g. pelvic dimensions) and tumour characteristics (e.g. rectal cancer local invasion and distance from the anal verge) [52–54]; hence requiring a preparation which accommodates these factors. The merger of anatomical models reconstructed from medical images and mental preparation introduces the possibility of patient-specific rehearsals for the above as well as other types of surgery.

Varying anatomy often complicates laparoscopic cholecystectomy (the procedure used in this study). Previous anatomical or radiological studies have categorised the relevant anatomical variations [55], which we can recreate in 3D anatomical models—models that can subsequently be used in combination with mental imagery to teach non-expert trainee surgeons. This is a relatively inexpensive method (3D reconstruction software is available as freeware and requires no specialist IT experience [56]), which could potentially boost surgical performance and ultimately lead to improved patient outcomes.
